# Towards an Interdisciplinary Consensual Measure of Social Participation: From Scoping Review to Clustering Measurement

**DOI:** 10.5334/irsp.854

**Published:** 2024-02-13

**Authors:** Jessica S. Morton, Bernard Rimé, Ginette Herman, David Bourguignon, Olivier Luminet

**Affiliations:** 1 Institut de recherche en sciences psychologiques, UCLouvain, Belgium; 2 Université de Lorraine, Metz, France; 3 Fund for scientific research (FRS-FNRS), Brussels, Belgium

**Keywords:** clusters, measurement, scoping review, social participation, large-scale survey

## Abstract

Given the current interest in social participation, this article focuses on existing measures that (1) include the four dimensions of formal social participation – breadth, intensity, duration, and engagement – identified by Bohnert et al. (2010) and (2) can be used in large-scale surveys. In Study 1, a scoping review conducted on three databases (PsychTest, PsychInfo, Sociological abstracts) identified 99 articles that included at least one measure of formal social participation. No measure met our two requirements. We therefore decided to design a new measure, which included an index based on six items to assess the four dimensions of the construct. Using clustering techniques, Study 2 identified social participation profiles based on the responses of 4,160 participants. Five clusters of social participation emerged: (0) absence, (1) passive, (2) low active, (3) medium active and (4) high active. Study 3 replicated these findings with a new sample (n = 3,956), thereby supporting the quality and replicability of the social participation measure by clustering. Coded as an ordinal categorical variable, the score lends itself to statistical analyses commonly performed on large-scale survey data. In this way, the Social Participation Index could meet the need for a standard tool that can be used in a multidisciplinary way.

Social participation refers to ‘people’s involvement in activities providing interactions with others in society or the community, content experts’ (Levasseur et al. 2010). Since the 1990s, social participation has been a fundamental concept, both in terms of scientific understanding and practical interventions. Émile Durkheim was among the first to recognise its importance in his research on suicide (1897) and religious rituals (1912). Since then, sociologists have explored social participation in various contexts, such as political action (e.g., social capital, Putnam, 1993) and social cohesion (e.g., Berkman et al., 2000). The fields of medicine and clinical practise have also shed light on the central role of participation in promoting health, benefiting both patients (e.g., Brady et al., 2011; Plug et al., 2008; Tobin et al., 2014) and older individuals (e.g., Ma et al., 2020; Townsend et al., 2021). In the realm of health and social psychology, efforts have been made to model the relationship between social participation and well-being, aiming to identify potential mediators and moderators (e.g., social support, Cohen, 1988; social identity, Jetten et al., 2012) and propose evidence-based interventions (e.g., Groups4Health, Haslam et al., 2019; social prescribing, Kellezi et al., 2021).

To expand research on social participation and gain a better understanding of how this intervention works (e.g., Costa et al., 2021) and for whom (e.g., Husk et al., 2020), it is crucial for researchers to establish a consensus on the concept. After conducting a scoping review, Levasseur et al. (2010) observed that numerous researchers had their own individual definitions of social participation, leading to confusion regarding its meaning. These varying approaches further complicated the matter. To promote standardisation in conceptualisation, the authors proposed an initial definition of social participation based on their content analysis. In their updated scoping review, Levasseur et al. (2022, p. 9) noted that the literature gradually was aligned with their suggested definition and that the focus was now more on the formal aspect of social participation, emphasising the importance of community life and shared spaces. In contrast, it does not capture informal social participation, which entails interactions with relatives, friends, and work colleagues in informal settings (e.g., Guillen et al., 2011; Lindström & Malmö Shoulder-Neck Study Group, 2006; Min et al., 2012).

While Levasseur et al. (2022) do emphasise the need to establish a consistent measurement of social participation aligned with the adopted definition, to the best of our knowledge, such a scale does not currently exist. This lack of a standardised scale hinders the ability to compare research findings in this area. This question is the focus of the present article. This is even more important as social participation is increasingly becoming a variable of interest in large-scale research. In line with the conceptual work of Levasseur et al. (2022), we conducted three studies. Study 1 entails a scoping review to assess the current practices for measuring social participation. Following a thorough content analysis, no measures were identified, either due to their normative usage or their failure to meet both quality and implementation criteria for large-scale studies (i.e., quantitative items applicable to large databases). Study 2 involves the development and validation of a measurement instrument specifically aligned with the concept of social participation, focussing on its formal dimension. Study 3 replicates the psychometric properties of the social participation index proposed in Study 2.

## Study 1: Scoping review

Bohnert et al. (2010) have pointed out that the complexity of measuring social participation has often been overlooked. A common practise is to compare participants in one or more social activities with non-participants, thus treating social participation as a dichotomous ‘all or nothing’ variable. In this way, respondents in the ‘participants’ group are assumed to have identical levels of participation. Significant differences in the intensity, duration and nature of their involvement are thus ignored. Building on previous studies (Mahoney et al., 2014; Roth et al., 2010; Weiss et al., 2013), Bohnert et al. (2010) argue that an accurate assessment of individuals’ participation in organised activities must consider the four dimensions of breadth, intensity, duration, and engagement. These authors define the breadth of participation (p. 580) as the number of different activity contexts in which individuals participate (i.e., sport, performing arts). Intensity of participation (p. 585) is defined as the frequency and time spent by an individual participating in a particular activity context. Duration (p. 590) is defined as the number of years an individual has participated in an organised activity. Finally, engagement (p. 593) is defined as a multidimensional concept encompassing different behaviours (e.g., attendance), emotions (e.g., enjoyment) and cognitions (e.g., acquisition of new skills). This four-dimensional conceptualisation of activities organised for young people, proposed by Bohnert et al. (2010), applies to any form of membership once social participation is limited to its formal dimension.

The scoping review addressed the following research question: Is there a measure of social participation in the current literature that encompasses all four of Bohnert et al.’s (2010) dimensions and can it be used in large-scale surveys?

### Method

A review of three databases, PsychTests, PsychInfo, and Sociological Abstracts, was conducted to cover two major fields in the study of social participation: psychology and sociology. To be included in the review, an article had to contain a measure of formal social participation. The search equation was limited to the term ‘social participation’ as a unique keyword in the title or abstract of the article. The inclusion of synonyms (e.g., social activity, volunteerism) was avoided to minimise noise in the search results. The review period was set from 1 January 2009 to 31 October 2021. This starting point was chosen to ensure that the work followed the scoping review of definitions undertaken by Levasseur et al. (2010). Articles that were not written in English or French were excluded.

#### Procedure

The article selection process was composed of three steps. First, all titles were reviewed to ensure that they included the term ‘social participation’. Any synonyms or broader concepts that included social participation (e.g. social capital) were retained so as not to exclude a relevant measure unnecessarily. Next, the abstract of each selected article was examined to determine whether the reported study included a measure of social participation. The inclusion criterion required a quantitative tool measuring any type of formal social participation (e.g., membership, extracurricular activities). Third, any measures of social participation were extracted from each article and analysed for content. To be included, social participation measures had to meet two criteria: (1) a consistent operationalisation with the definition of formal social participation as membership in a structured social group, and (2) easily applicable item formats for large-scale surveys. If either criterion was not met, the measure was removed from our selection. As for coding, for each stage we worked in three rounds: (1) full analysis by the first author; (2) discussion to refine the inclusion and exclusion criteria with the research team; (3) coding by the first author with random checking by another member of the team.

### Results

Of the 921 items retrieved from the databases, 10 were duplicates. Of the remaining 911 items, 491 were excluded because their titles did not contain the concept of social participation or its equivalent, or because their titles referred to restricted social participation (e.g., physical disability) instead of social participation in its broadest sense. Of the remaining 420 articles, 190 were excluded because they did not mention any measures of social participation in their abstract. Of the remaining 230 articles, 25 could not be accessed and 106 were excluded because they did not use a formal measure of social participation. The final selection consisted of 99 articles reporting on the use of the searched measure (see Table 1). They are grouped according to the source of the social participation measure used in the reported study. Five categories of sources were distinguished: (a) scales from previous studies (n = 17 different measures/30 articles), (b) items from large-scale surveys (n = 29/30), (c) items from a previous study (n = 8/8), (d) scales created by the authors of the article themselves (n = 12), and (e) authors created one or more items without giving them a specific name (n = 19).

**Table 1:** Articles from the scoping review grouped according to the social participation measure used.


#	REVIEWED ARTICLES	#	USED MEASURES OF SOCIAL PARTICIPATION	BREADTH	INTENSITY	DURATION	ENGAGEMENT

*Scales from previous studies*							

**1–11**	Bult et al., 2013; Bult et al., 2014; Clarke et al., 2012; Dahan-Olielet al., 2014a; Dahan-Oliel et al., 2014b; Kreider etal., 2015; Kreider et al., 2016; McMullan et al., 2012; Shields et al., 2014; Shields et al., 2015; Shikako-Thomaset al., 2013; Van Wely et al., 2014	1	Children’s Assessment of Participation and Enjoyment and Preferences for Activities of Children (CAPE, King et al., 2007)	1	1	0	0

**12**	Thraen-Borowski et al., 2013	2	Community Healthy Activities Model Program for Seniors (CHAMPS, Stewart et al., 2001)	1	0	0	0

**13**	Biddle et al., 2019	3	Community Healthy Activities Model Program for Seniors (different operating)	1	1	1	0

**14**	McLean et al., 2012	4	Community Integration Questionnaire, social integration scale (Barnea et al., 2004)	1	1	0	0

**15–16**	Julien etal., 2013; Richard et al., 2013	5	Elderly Activity Inventory Questionnaire (Lefrançois et al., 2001)	1	1	0	0

**17–18**	Alma et al., 2011, 2012	6	ICF Measure of Participation and ACTivities – Screener (IMPACT-S, Post, 2008)	1	0	0	0

**19**	Tsaiet al., 2015	7	Index of extended ties (Berkman, 1977)	1	1	0	0

**20**	James et al., 2011	8	Late-life social activity (Bennett et al., 2005)	1	1	0	0

**21**	Mikkola et al., 2015	9	Life-Space Mobility in Old Age (LISPE – Finland, Rantanen et al., 2012)	1	1	0	0

**22**	Nyqvist et al., 2012	10	Measuring social capital (Stone, 2001)	1	0	0	1

**23**	Flynn et al., 2018	11	Participation Assessment with Recombined Tools-Objective (PART-O, Whiteneck et al., 2011)	1	1	0	0

**24**	Rutenfrans-Stupar et al., 2019	12	Participation Ladder (Van Gentet al., 2008)	1	0	0	0

**25**	Bielak et al., 2012	13	RIASEC Activity List (Parslow, 2006)	1	0	0	0

**26**	Bisung et al., 2014	14	Social Capital Assessment Tool (SOCAT, Krishna & Shrader, 2000)	0	0	0	0

**27–28**	Glei et al., 2012; Marcus et al., 2015	15	Social Network Index (SNI, Ford et al., 2006)	0	1	0	0

**29**	Chang et al., 2015	16	Temple University Community Participation Measure (CPDM, Wong et al., 2007)	0	1	0	0

**30**	Brown et al., 2016	17	Victoria Longitudinal Study (VLS) Activity Lifestyle Questionnaire, 1986, 1993	1	1	0	0

*Items from large-scale surveys*							

**31**	Buffel et al., 2014	18	Belgian Ageing studies (BAS – Belgium), 2004	1	0	0	1

**32**	Fiorillo et al., 2020	19	British Household Panel Survey (BHPS), 1991–1995	0	0	0	1

**33–34**	Winters & Rundlett, 2015; Lindström & Giordano, 2016	20	British Household Panel Survey (BHPS), 1991–2009	0	0	0	0

**35**	Wang et al., 2019	21	China Health and Retirement Longitudinal Study (CHARLS), 2011, 2013, 2015	1	1	0	0

**36**	Pan & Chee, 2020	22	China Health and Retirement Longitudinal Study (CHARLS), 2011, 2015	1	1	0	0

**37**	Haghighat & Knifsend, 2019	23	Education Longitudinal Study, 2002	1	0	0	0

**38**	Cruwyset al., 2013	24	English Longitudinal Study of Aging (ELSA), 2002–2203, 2011–2012	1	0	0	0

**39**	Kouvonen et al., 2012	25	English Longitudinal Study of Aging (ELSA), 2004–2005, 2008–2009	1	0	0	0

**40**	Valentova, 2016	26	European Values Study (EVS, Luxembourg), 2008	1	0	0	0

**41**	Peng et al., 2019	27	General Health Professions Student Survey (GHPSS – China), 2013	1	1	0	0

**42**	Hajeket al., 2017	28	German Srydy on Ageing, Cognition and Dementia in Primary Care Patients (AgeCoDe), 2003–2004, 2011–2012	0	1	0	0

**43**	Chen et al., 2019	29	Health and Retirement Study (HRS – US), 2010, 2012	1	1	0	0

**44**	Rosso et al., 2013	30	Household Health Survey (HHS – US), 1994	0	1	0	0

**45**	Yoshida et al., 2020	31	Japan Gerontological Evaluation Study (JAGES), 2003–2004, 2006–2007, 2010–2011, 2013, 2016	1	1	0	0

**46**	Hikichiet al., 2020	32	Japan Gerontological Evaluation Study (JAGES), 2010	0	1	0	0

**47**	Nishio et al., 2021	33	Japan Gerontological Evaluation Study (JAGES), 2010, 2013	1	1	0	0

**48**	Choi etal., 2015	34	Korean Longitudinal Study of Aging (KloSA), 2006, 2008, 2010	1	0	1	0

**49**	Moore & Carpiano, 2020	35	Montreal Neighboorhood Networks and Healthy Aging (MoNNET), 2008, 2010, 2012–2013	0	0	1	0

**50**	Kim et al., 2017	36	National Health and Aging Trends Study (NHATS – US), 2010	1	0	0	0

**51**	Amagasaet al., 2017	37	National Health and Nutrition Survey (NHNS), 2006	0	1	0	0

**52**	Chen et al., 2016	38	National Social Life, Health, and Aging Project (NSHAP – US), 2005–2006, 2010–2011	1	1	0	0

**53**	Lounds Taylor et al., 2017	39	National Survey of Families and Household (Bumpass & Sweet, 1987)	1	1	0	0

**54**	Gonzalez et al., 2020	40	Social Networks and Social Resources Survey, 30 countries around the world (ISSP), 2017	1	1	0	0

**55**	Saïas et al., 2012	41	Survey of Health, Ageing and Retirement in Europe (SHARE, Börsch-Supan & Jürges, 2005), 2006–2007	0	0	0	0

**56**	Santini et al., 2020	42	Survey of Health, Ageing and Retirement in Europe (SHARE), 2011, 2013, 2015	1	1	0	0

**57**	Niedzwiedz et al., 2016	43	Survey of Health, Ageing and Retirement in Europe (SHARE), 2013	1	1	0	0

**58**	Witvorapong, 2018	44	Surveys of Older Persons in Thailand, 2008	0	0	0	0

**59**	Chiao, 2019	45	Taiwan Longitudinal Study on Aging (TLSA), 1993, 1996, 1999, 2003, 2007	0	0	0	0

**60**	Ihle et al., 2016	46	Vivre-Leben-Vivere survey (VLV – Switzerland), 2011, 2012	1	0	0	0

*Items from a previous study*							

**61**	Yu et al., 2015	47	Bain & Hicks, 1998	0	0	0	0

**62**	Ball et al., 2010	48	Baum, 1999	1	1	0	0

**63**	Sharp et al., 2015	49	Eccles et al., 2003	0	0	0	0

**64**	Luo & Menec, 2018	50	Foxton & Jones, 2011	1	0	0	0

**65**	Habibov & Weaver, 2014	51	Harper, 2002	0	0	0	0

**66**	Leedahl et al., 2018	52	Narayan & Cassidy, 2001	0	0	0	0

**67**	Meng & Chen, 2014	53	Norstrand & Xu, 2012	1	1	0	0

**68**	Ang, 2018	54	Veroff et al., 1981	0	1	0	0

*Scales created by the authors of the study*							

**69**	Aoki et al., 1996	55	21-item Total Social Activity Measure	1	1	0	0

**70**	Lancee & Van de Werfhorst, 2012	56	Civic and Social Participation Survey	1	0	0	0

**71**	Rinkus et al., 2016	57	Civic, Community, and Social Participation Measures	0	1	1	0

**72**	Guillen et al., 2011	58	Measurement of Social Participation	1	0	0	1

**73**	Tuffrey et al., 2013	59	Questionnaire of Young People’s Participation (QYPP)	1	1	0	0

**74**	Moreno-Jiménez et al., 2013	60	Scale participation (SCAP)	1	1	0	0

**75**	Li et al., 2011	61	Social activity questionnaire	1	0	0	0

**76**	Thomas, 2011	62	Social engagement composite measure items	1	1	0	0

**77**	Infurna et al., 2011	63	Social Participation Measure	1	1	0	0

**78**	Zhang & Zhang, 2015	64	Social Participation Questionnaire	0	1	0	1

**79**	Densley et al., 2013	65	Social Participation Questionnaire (SPQ)	1	1	0	0

**80**	Howrey& Hand, 2019	66	Social participation scale (SoPart-30)	1	1	0	0

*Items created by the authors of the study*							

**81**	Aidaet al., 2012	67		0	0	0	0

**82**	Calvin et al., 2015	68		1	0	0	0

**83**	Chappell & Funk, 2010	69		0	0	0	0

**84**	Child & Lawton, 2019	70		0	1	0	0

**85**	Donnelly & Hinterlong, 2010	71		0	1	0	0

**86**	Dury et al., 2021	72		1	1	0	1

**87**	Ejiri et al., 2019	73		1	0	0	0

**88**	Eriksson & Ng, 2015	74		0	1	0	0

**89**	Giordano & Lindstrom, 2010	75		0	0	0	0

**90**	Hsu & Chang, 2015	76		0	0	0	0

**91**	Hughes etal., 2013	77		1	1	0	0

**92**	Jerliu et al., 2014	78		0	0	0	0

**93**	Legh-Jones & Moore, 2012	79		0	0	0	0

**94**	Otsuka et al., 2018	80		1	1	0	0

**95**	Pavlova et al., 2014	81		0	1	0	0

**96**	Takagi et al., 2016	82		1	0	0	0

**97**	Tomiokaet al., 2015	83		1	0	0	0

**98**	Tong et al., 2011	84		0	1	0	0

**99**	Yokobayashi et al., 2017	85		1	1	0	0

			**Total**	**54**	**46**	**4**	**6**

Note. To find the full reference of the used measures of social participation, the reader should refer to the related reviewed article.							


This brings the total number of different social participation measurement tools to 85. For each of them, Table 1 indicates whether or not the measure includes each of the four dimensions mentioned by Bohnert et al. (2010). Thus, the dimensions were coded 1 if the item measures quantity for the breadth dimension, frequency for the intensity dimension, more than 12 months for duration, and active participation for engagement.

The objective of this scoping review was to identify a measure of social participation which encompassed all four of Bohnert et al.’s (2010) dimensions. The review revealed that, among the 85 instruments analysed, 54 (63.5%) included a measure of breadth, 47 (55.3%) of intensity, 4 (47%) of duration and 6 (7.1%) of engagement. Of the 85 instruments reviewed, 15 (17.6%) did not include any of the Bohnert et al. dimensions, 31 (36.4%) assessed only one, 37 (43.5%) assessed two and 2 (2.4%) assessed three. None addressed all four dimensions. The literature we reviewed did not reveal the instrument we had been looking for.

### Discussion

This scoping review found that none of the measures identified considered together the four dimensions of social participation identified by Bohnert et al. (2010) as essential. Furthermore, the review revealed the surprising abundance and diversity of measures adopted by different studies. We therefore concluded that there is a need to develop a measure of social participation that considers all four dimensions.

## Study 2

The aim of this second study has been to develop and test an instrument to measure social participation that meets the following three requirements: (a) include the four dimensions of social participation proposed by Bohnert et al. (2010); (b) be composed of a limited number of quantitative items to avoid unnecessarily lengthy surveys and facilitate data processing; and (c) provide a score that can be used for conventional statistical analyses (e.g., comparison, regression, modelling). These quality and feasibility criteria are in line with the needs of researchers who want to include a measure of social participation in a large-scale survey.

To fulfil these various requirements, we opted for a clustering measure of social participation. This multivariate analysis technique involves an unsupervised exploratory approach. It synthesises a set of items by identifying a limited number of natural groups within the sample without any transformation of the initial data. According to James et al. (2013), clustering can be a very useful and valid statistical tool if applied correctly. Indeed, these authors highlight the significant impact that small decisions (e.g., choice of clustering method, use of standardised scores) can have on the results. For this reason, these authors recommend carrying out several clustering solutions, varying the choices and examining all the results to identify the trends that emerge in a consistent way. Consequently, our clustering analyses were carried out using IBM SPSS Statistics (Version 28), which offers three different clustering methods: hierarchical cluster analysis, K-means and TwoStep analyses. This combination of hierarchical and non-hierarchical procedures is recommended by Hair et al. (2010). They were applied in cascade to produce consistent and easily interpretable profile results. The procedure has been detailed in the results section to make it easier for interested researchers to use this new measure.

### Operationalising the four dimensions of social participation

Our operationalisation of social participation considers both Bohnert et al.’s (2010) recommendations and the limitations noted in the prior scoping review.

To assess *breadth*, Bohnert et al. (2010) suggested including the total number of different activity contexts in which activities are carried out, as well as activity dispersion (i.e., the extent to which participation is concentrated in one or multiple domains). To measure the breadth dimension, the use of predefined lists of social activities (e.g., swimming, dancing, playing cards) was suggested by Bohnert et al. (2010). From a practical point of view, however, the creation of such a list is often too specific to be directed to a sample of the general population (regardless of age, culture, illness, etc.). To overcome this issue, Guillen et al. (2011) have used universal themes on which social groups develop (e.g., sport, culture, trade union, religion, environment, humanitarian) to reach a relatively broad audience. In other words, the sum of the themes ticked in the list would correspond to the number of activities carried out in different social groups (i.e., activity dispersion).

For *social engagement*, Bohnert et al. (2010) proposed a qualitative format intended to assess the degree of engagement in social participation or to use an experience sampling method. Unfortunately, these two proposals cannot be applied in large-scale surveys. Guillen et al. (2011) have proposed an alternative appropriate for large surveys. It consists of asking the respondent about their social role (i.e., none, member, participation, and volunteer) for each of the 12 social activity themes listed. However, such a cross-tabulated format (breadth and engagement) is not desirable for researchers interested in analysing the dimensions separately. In order to counter this problem, we asked respondents to reply to two distinct items: by selecting (a) all the themes in the list for which they are invested in a social group (breadth item) and (b) all the social roles assumed independently of the different social groups with which they are associated (engagement item). Another limitation of the Guillen et al. (2011) proposal lies in the ambiguity of the participation labels (such as ‘active participation’) and their position in the list. A member who actively participates in the organisation of a social group is more active than a member who occasionally provides some help as a volunteer. For this reason, two other changes have been made to the initial proposal by Guillen et al. (2011): (a) the ‘organiser’ label is clearer than participation and has been preferred in our measure, and (b) by switching the ordering of social roles from *organiser then volunteer* to *volunteer then organiser*, the degree of social engagement is better reflected.

*Intensity* refers to (a) the frequency with which the individual participates in social groups and (b) the time dedicated to social participation. Bohnert et al. (2010) advise against using the number of activities as an indicator of intensity. According to these authors, this type of item does not accurately capture intensity because the time allocated to social participation varies greatly from one type of activity to another (e.g., weekly sports training vs. one-off help at the annual neighbours’ party). The authors prefer to ask an open-ended question to assess intensity, but this type of question is not applicable to a large-scale survey. From a practical point of view, the Likert scale format seems most appropriate as the response choices can take the different temporalities for both frequency (e.g., daily, several times a week, monthly, yearly) and the hourly volume of social participation (e.g., time engaged in each activity per week, Biddle et al., 2019) into account. However, asking participants to make such a calculation is risky. The calculation can be complicated depending on the period investigated (e.g., per year) and the reality of daily life (e.g., two to three meetings with the social group of one and a half hours per week). The more complicated this calculation is for respondents (e.g., three to four hours/week * 52 weeks), the higher the probability of drop-out or error. To overcome this difficulty, a monthly hourly volume item offers a temporality that is easy to calculate for frequent activities (e.g., three to four hours/week = 12 to 16 hours/month) as well as for occasional activities (e.g., less than once a month).

To evaluate *duration*, Bohnert et al. (2010) have proposed that researchers calculate the number of years spent doing the activity or opt for a longitudinal design. In both cases, the Likert scale format is once again appropriate. Likert labels proposing ranges of periods (e.g., less than six months, three to five years, more than 10 years) make it easy to define whether the duration is short-, medium-, or long-term.

### Overview of Study 2

In accordance with the James et al.’s (2013) recommendations, the quality of the identified clusters was assessed in the following way: (a) by comparing the clusterings to check for a certain consistency between the tested solutions, and (b) by first testing clusterings with averaged scores and then standardised scores. For both types of scores, the clusters were systematically interpreted using the social roles variable (i.e., no participation, member, volunteer, and/or organiser) to ensure that the clusters reflected the degree of social participation.

It was required that the expected social participation score be an ordinal categorical variable (a) composed of a limited number of clusters to facilitate comparison analyses and (b) whose interpretation reflected the degree of social participation (from not at all to very high) so it could be used in a Boolean format in regression and modelling analyses.

Finally given that we obtained the expected results, we thought it would be worthwhile re-testing this measure with a second sample. To do this, the initial sample (N = 8,116 respondents) was randomly divided into two sub-samples (e.g., Heller et al., 2009). This procedure made it possible to obtain two comparable samples on which to replicate the same analyses and allowed us to conclude that the method used was robust.

### Method

#### Procedure

Our data comes from a survey conducted in collaboration with the Mutualité Chrétienne. It is the Belgium’s largest social security and health insurance fund. It offers its 4.6 million members benefits and services based on solidarity and fights for quality care accessible to all. They agreed to include items measuring social participation in their survey on the benefits of volunteering for health and well-being. For the present study, only these items from the survey were used. The questionnaire was distributed online to all affiliates on their mailing list. Their legal department ensured that the ethical aspects were respected.

#### Participants

The first sub-sample consisted of 4,160 respondents with a mean age of 59.23 years (SD = 15,390; min = 19; max = 92), 55.6% of whom were women.

#### Measures

The social participation index was composed in six items measuring social participation (see supplementary material available at https://osf.io/vumdw/?view_only=ffdac17d1e7e470aa78d699ee9b27cc6).

##### Membership

First, respondents reported whether they were involved or not in a club, organisation, or association (item #1). Second, depending on the previous response, respondents were redirected differently. Those who replied that they were not members of formal social participation organisations (N = 1359) were directed to the next section of the survey and received a coded response of 0 for the items measuring social participation. Those who mentioned being involved in social groups were asked five questions intended to delve deeper into their social participation.

##### Breadth of social participation

We used a list (item #2) of 17 types of social groups: sport, culture, youth, training & education, health & well-being, disability, elderly, women, social & political, religion & philosophy, poverty, folklore, humanitarian, environment, leisure & holidays, neighbourhood, and others. Examples of social groups were provided for each theme (e.g., health & well-being: Red Cross, home or hospital volunteering, tele-hosting). Multiple responses were permitted. The score for the breadth variable corresponded to the sum of the different social groups in which respondents were involved.

##### Social engagement

This item (#3) posed the following question: ‘What does your participation in the club, organisation, or association listed in the previous question involve?’ Participants could select one or more of the following: (#3a) I am a member, I participate in activities; (#3b) I volunteer, I carry out some activities on a voluntary basis; and (#3c) I am part of the management/administration, I organise activities.

##### Intensity of social participation

The intensity of social participation was measured according to (a) the frequency with which respondents attended their social groups and (b) the time spent on these social activities. On the one hand, frequency was assessed with the following item (#4): ‘For each of the three social roles, how often do you participate in activities organised by the clubs, organisations or associations to which you belong?’ Six-point response scales were used for each social role (#4a; #4b; #4c): 1 = several times a week; 2 = once a week; 3 = several times a month; 4 = once a month; 5 = several times a year; 6 = once a year; and, for respondents with no social participation, 0 = never. Hourly volume was assessed by the following item (#5): ‘For each of the three social roles, how many hours per month do you participate in activities organised by the clubs, organisations or associations to which you belong?’ Respondents rated each social role (#5a; #5b; #5c) on an 8-point scale anchored with 1 = less than one hour; 2 = 1–5 hours; 3 = 6–10 hours; 4 = 11–20 hours; 5 = 21–30 hours; 6 = 31–45 hours; 7 = 46–60 hours; 8 = more than 61 hours per month; and 0 = 0 hours.

##### Duration of social participation

This item (#6) asked ‘How long have you been involved in your club, organisation, or association?’ Unlike the previous items, this one was not broken down into the three social roles. Duration was rated on a 7-point scale anchored with 1 = less than 6 months, 2 = 6–12 months, 3 = 1–2 years, 4 = 3–5 years, 5 = 5–10 years, 6 = more than 10 years, and 0 = not currently.

The items measuring the four dimensions of social participation were introduced into the clustering analyses without any alterations. Afterwards, to facilitate the interpretation of the identified clusters, the three social engagement sub-items were combined to create the social roles variable.

##### Social Roles

In the social engagement question, respondents could report taking on several roles in their social groups. Responses were recoded into ‘no social engagement’ (No = 0), ‘member’ (M = 1), ‘volunteer’ (V = 2) and/or ‘organiser’ (O = 4). Assigning a score of 4 to the organisers allowed us to create a new categorical variable by adding the responses to the three sub-items of social engagement. The MVO variable breaks the degree of social engagement down into eight categories: 0 = no social engagement, 1 = member, 2 = volunteer, 3 = member & volunteer, 4 = organiser, 5 = member & organiser, 6 = volunteer & organiser, and 7 = member & volunteer & organiser.

### Results

With this first sub-sample, we aimed to observe natural groups of social participation that could be easily interpreted and for which the score would be an ordinal categorical variable. To achieve this, multivariate clustering analyses were first carried out with mean scores and then standardised scores.

#### Clustering using mean scores

##### Data consistency

**Figure 1:**
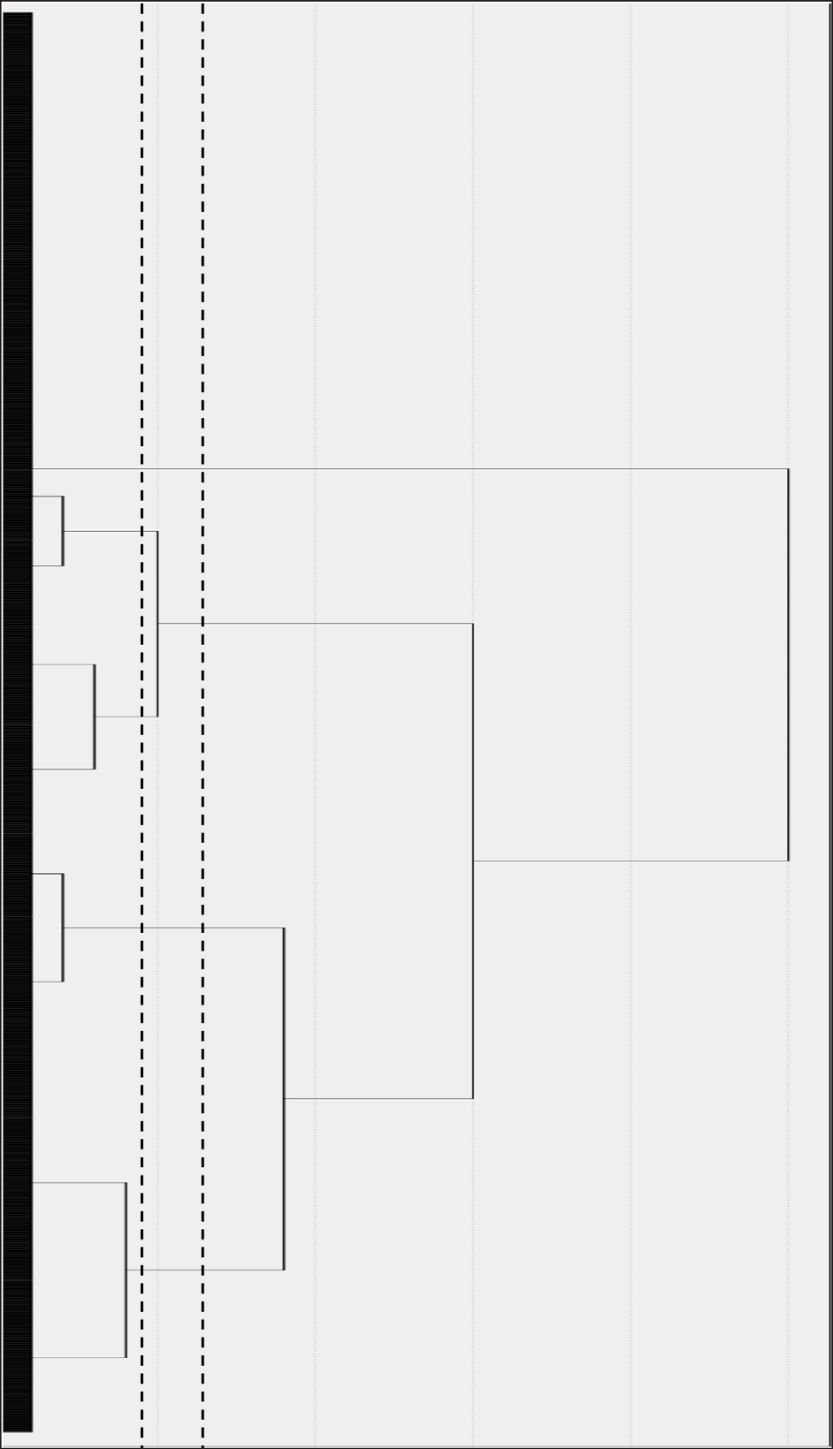
Hierarchical cluster analysis dendrogram with the first sub-sample. *Note*. Dashed lines refer to the spots used to select the number of clusters to be tested. The length of the horizontal lines indicates the distance between the elements. The longer the line, the more heterogeneous the cluster.

In line with the Hair et al.’s (2010) recommendations, the dendrogram from the hierarchical cluster analysis provides an initial visual exploration of the data to determine the number of clusters to fix in the non-hierarchical analyses. For this first sub-sample, the dendrogram proposed two solutions of 4 or 5 clusters (see Figure 1). These solutions were tested with the non-hierarchical K-Means and TwoStep methods provided by SPSS.

To assess the consistency of the results, the four new solutions had to (1) group respondents with equivalent social roles together and (2) propose similar clusterings from one solution to the other. Table 2 presents the clusterings of the four solutions tested (i.e., 4 and 5 clusters; K-means/TwoStep method) with the mean scores.

**Table 2:** Description of the profiles provided by the four solutions (4 and 5 clusters * K-means and TwoStep) tested using mean scores from the first sub-sample.


	ORDINAL CODING	FIXED AT 4 CLUSTERS				FIXED AT 5 CLUSTERS				
	
		0	1	2	3	0	1	2	3	4

	INTERPRETATION	NO	PASSIVE	LOW	HIGH	NO	PASSIVE	LOW	MEDIUM	HIGH

**K-means**	**Breadth**	0	2	2	2	0	2	2	2	2

	**Engagement M**	0	1	0	1	0	1	0	0	1

	**Engagement V**	0	0	2	1	0	0	2	0	2

	**Engagement O**	0	0	0	4	0	0	0	4	4

	**Intensity Fre M**	0	5	1	2	0	5	1	2	3

	**Intensity Fre V**	0	0	4	2	0	0	4	0	4

	**Intensity Fre O**	0	0	0	4	0	0	0	4	4

	**Intensity Vol M**	0	3	1	1	0	3	1	1	2

	**Intensity Vol V**	0	0	3	1	0	0	3	0	3

	**Intensity Vol O**	0	0	0	3	0	0	0	3	3

	**Duration**	0	5	5	5	0	5	5	5	5

	**N**	**1335**	**1027**	**782**	**1016**	**1330**	**1012**	**772**	**619**	**427**

**TwoStep**	**Breadth**	0.03	1.81	2.00	2.14	0.03	1.81	2.00	2.07	2.21

	**Engagement M**	0.00	0.98	0.48	0.52	0.00	0.98	0.48	0.48	0.58

	**Engagement V**	0.00	0.02	2.00	0.87	0.00	0.02	2.00	0.00	2.00

	**Engagement O**	0.00	0.04	0.03	4.00	0.00	0.02	0.00	4.00	4.00

	**Intensity Fre M**	0.00	4.43	2.03	2.21	0.00	4.43	2.04	2.06	2.43

	**Intensity Fre V**	0.00	0.00	3.87	1.71	0.00	0.00	3.87	0.00	3.97

	**Intensity Fre O**	0.00	0.01	0.00	3.55	0.00	0.00	0.00	3.53	3.51

	**Intensity Vol M**	0.00	2.77	1.27	1.45	0.00	2.77	1.28	1.29	1.66

	**Intensity Vol V**	0.00	0.00	2.76	1.28	0.00	0.00	2.75	0.00	2.98

	**Intensity Vol O**	0.00	0.00	0.00	2.97	0.00	0.00	0.00	2.89	3.00

	**Duration**	0.08	4.73	4.76	5.37	0.08	4.72	4.75	5.37	5.37

	**N**	**1317**	**839**	**943**	**1061**	**1317**	**862**	**936**	**606**	**466**


*Note*. N = 4160. Fre = frequency; Vol = hourly volume per month; M = member; V = volunteer; O = organiser. The scores shown are the average of the respondents in the same cluster.

The four solutions tested resulted in similar clusters, suggesting a certain consistency in the data. We systematically found four clusters: a *no social participation* cluster; a cluster that we named *passive* social participation (i.e. as a member); and two clusters that fell under the category *active* social participation and which were classified as *low* (i.e. as a member and volunteer) or *high* (as a member, volunteer and organizer). The identification of a fifth cluster made it possible to divide the higher cluster in two and thus obtain an intermediate level of active social participation (i.e., as a member & organiser). A five-cluster solution provided a more sensitive categorical variable than a four-cluster solution, suitable for comparison, regression, and modelling analyses. For this reason, it was preferred to a four-cluster solution.

##### Cluster interpretation

Interpreting the profiles using the social roles variable made it possible to (a) assess the extent to which the clusters reflected the degree of social participation and (b) identify the most consistent solution between the K-means and TwoStep methods. Table 3 presents the frequencies of social roles (MVO) for each cluster obtained with the two non-hierarchical methods.

**Table 3:** Frequencies of social roles for each cluster provided by K-means and TwoStep methods using mean scores from the first sub-sample.


SOCIAL ROLES		NO	M	V	M + V	O	M + O	V + O	M + V + O	TOTAL

**K-means**	**Cluster 1**	**1304**	18	6	0	1	1	0	0	1330

	**Cluster 2**	25	**790**	0	175	0	21	0	1	1012

	**Cluster 3**	0	0	**478**	**286**	0	0	6	2	772

	**Cluster 4**	0	0	0	0	**312**	**275**	16	16	619

	**Cluster 5**	0	0	0	0	0	0	**177**	**250**	427

	**Total**	1329	808	484	461	313	297	199	269	4160

**TwoStep**	**Cluster 1**	**1314**	2	0	0	1	0	0	0	1317

	**Cluster 2**	15	**806**	0	10	0	4	0	0	835

	**Cluster 3**	0	0	**484**	**451**	0	0	1	0	936

	**Cluster 4**	0	0	0	0	**312**	**293**	1	0	606

	**Cluster 5**	0	0	0	0	0	0	**197**	**269**	466

	**Total**	1329	808	484	461	313	297	199	269	4160


*Note*. No = no social participation; M = member; V = volunteer; O = organiser. Figures in bold refer to a consistent distribution of respondents based on their social roles.

The correspondence rates between the social roles variable (MVO) and the cluster distribution were 93.08% for the K-means solution and 99.18% for the TwoStep solution. Furthermore, a comparison between these two solutions showed that 93.89% of respondents were distributed in a similar way regardless of the method used (see Table 4).

**Table 4:** Cross-tabulation of the K-means and TwoStep solutions fixed at 5 clusters using mean scores from the first sub-sample.


K-MEANS	TWOSTEP					TOTAL

	NO	PASSIVE	LOW	MEDIUM	HIGH	

**no**	1307	16	6	1	0	1330

**passive**	10	819	**165**	17	1	1012

**low**	0	0	765	0	7	772

**medium**	0	0	0	588	**31**	619

**high**	0	0	0	0	427	427

**Total**	1317	835	936	606	466	4160


*Note*. No = no social participation; passive = passive social participation; low = low active social participation; medium = medium active social participation; high = high active social participation. Figures in bold refer to a different distribution of respondents according to the clustering method used.

Among the differences observed, 165 respondents were distributed in the *passive* cluster with the K-means method and in the *low* cluster with the TwoStep method, while 31 respondents were distributed in the *medium* and *high* clusters respectively. We decided to look more closely at what kind of social participation these respondents had. Table 5 presents the descriptive of the variables measuring social participation for these 196 respondents distributed differently according to the clustering method used.

**Table 5:** Description of profiles of 165 and 31 respondents grouped differently according to the clustering method used.


DIMENSIONS	N = 165		N = 31	
	
	*M*	*SD*	*M*	*SD*

**Breadth**	2.37	1.06	1.94	1.06

**Engagement M**	1.00	0.00	0.52	0.51

**Engagement V**	2.00	0.00	2.00	0.00

**Engagement O**	0.00	0.00	4.00	0.00

**Intensity Fre M**	4.87	1.13	1.65	1.94

**Intensity Fre V**	2.21	0.95	1.39	0.92

**Intensity Fre O**	0.00	0.00	2.61	1.28

**Intensity Vol M**	3.18	1.34	0.97	1.22

**Intensity Vol V**	1.99	0.87	1.16	0.93

**Intensity Vol O**	0.00	0.00	2.06	1.77

**Duration**	4.91	1.23	5.39	0.92


*Note*. Fre = frequency; Vol = hourly volume per month; M = member; V = volunteer; O = organiser. The scores shown are the average of the selected respondents.

An analysis of these profiles indicated that the 165 respondents were involved in some form of social participation corresponding to the social roles of *member & volunteer* and that the 31 respondents were involved in some form of social participation corresponding to the social roles of *member & volunteer & organiser*. These profile descriptions corresponded to the *low* and *high* active social participation clusters respectively. Thus, the solution obtained with the TwoStep method produced more homogeneous clusters than those obtained with the K-means method.

Although the two solutions resulted in very similar clusterings, a closer examination of the profiles indicated that a measure involving five clusters obtained from the mean scores with the TwoStep solution was a more accurate option.

#### Clustering using standardised scores

Researchers interested in inter-cluster comparisons may wish to carry out intra-sample comparison analyses. In this case, they will need to use standardised scores. As James et al. (2013) have pointed out, the format of the score can influence the clustering results. For this reason, the 5-cluster fixed solutions obtained with the K-means and TwoStep methods were reproduced using z-scores instead of mean scores.

##### Data consistency

It was expected that the two solutions (i.e., K-means and TwoStep) used (1) would group respondents with similar social roles together and (2) would propose clusters equivalent to those obtained with the mean scores. Table 6 presents the two clusterings set at 5 clusters obtained with the K-means and TwoStep methods from the standardised scores obtained with the first sub-sample.

**Table 6:** Description of the profiles provided by the two solutions (K-means and TwoStep) tested using z- scores from the first sub-sample.


	ORDINAL CODING	0	1	2	3	4

	INTERPRETATION	NO	PASSIVE	LOW	MEDIUM	HIGH

**K-means**	**Breadth**	–1.02	0.36	0.47	0.55	0.66

	**Engagement M**	–0.88	1.12	0.00	0.06	0.29

	**Engagement V**	–0.72	–0.53	1.39	–0.70	1.39

	**Engagement O**	–0.59	–0.55	–0.56	1.69	1.69

	**Intensity Fre M**	–0.81	1.11	–0.05	0.03	0.23

	**Intensity Fre V**	–0.64	–0.57	1.32	–0.64	1.30

	**Intensity Fre O**	–0.53	–0.52	–0.53	1.56	1.57

	**Intensity Vol M**	–0.75	1.02	–0.07	0.01	0.29

	**Intensity Vol V**	–0.60	–0.53	1.19	–0.60	1.28

	**Intensity Vol O**	–0.50	–0.48	–0.50	1.42	1.52

	**Duration**	–1.29	0.51	0.52	0.76	0.76

	**N**	**1331**	**903**	**878**	**597**	**451**

**TwoStep**	**Breadth**	–1.04	0.33	0.49	0.54	0.65

	**Engagement M**	–0.89	1.09	0.08	0.09	0.27

	**Engagement V**	–0.72	–0.69	1.39	–0.71	1.39

	**Engagement O**	–0.59	–0.58	–0.59	1.69	1.69

	**Intensity Fre M**	–0.81	1.06	0.05	0.06	0.21

	**Intensity Fre V**	–0.64	–0.64	1.24	–0.64	1.29

	**Intensity Fre O**	–0.53	–0.53	–0.53	1.53	1.52

	**Intensity Vol M**	–0.75	0.96	0.04	0.04	0.27

	**Intensity Vol V**	–0.60	–0.60	1.12	–0.60	1.27

	**Intensity Vol O**	–0.50	–0.50	–0.50	1.40	1.47

	**Duration**	–1.31	0.51	0.52	0.76	0.76

	**N**	**1317**	**834**	**936**	**607**	**466**


*Note*. N = 4160. Fre = frequency; Vol = hourly volume per month; M = member; V = volunteer; O = organiser. The scores shown are the average of the respondents in the same cluster.

Rather similar groups were identified in the two new solutions tested with the z-scores. There was systematically one *no* social participation cluster, one *passive* social participation (i.e., as a member) cluster and three *active* social participation (i.e., *low*: as a volunteer; *medium*: as an organiser; and *high*: as a member, volunteer, and organiser) clusters. The counts were relatively similar across the solutions (K-means vs. TwoStep) and consistent with those obtained from the mean scores.

##### Cluster interpretation

The correspondence rates between the social roles variable (MVO) and the cluster distribution were 97.14% for the K-means solution and 99.21% for the TwoStep solution (see Table 7).

**Table 7:** Frequencies of social roles for each cluster provided by K-means and TwoStep methods using z-scores from the first sub-sample.


SOCIAL ROLES		NO	M	V	M + V	O	M + O	V + O	M + V + O	TOTAL

**K-means**	**Cluster 1**	**1327**	3	0	0	1	0	0	0	1331

	**Cluster 2**	2	**805**	0	78	0	18	0	0	903

	**Cluster 3**	0	0	**484**	**383**	0	0	7	4	878

	**Cluster 4**	0	0	0	0	**312**	**279**	4	2	597

	**Cluster 5**	0	0	0	0	0	0	**188**	**263**	451

	**Total**	1329	808	484	461	313	297	199	269	4160

**TwoStep**	**Cluster 1**	**1314**	2	0	0	1	0	0	0	1317

	**Cluster 2**	15	**806**	0	10	0	3	0	0	834

	**Cluster 3**	0	0	**484**	**451**	0	0	1	0	936

	**Cluster 4**	0	0	0	0	**312**	**294**	1	0	607

	**Cluster 5**	0	0	0	0	0	0	**197**	269	466

	**Total**	1329	808	484	461	313	297	199	**269**	4160


*Note*. No = no social participation; M = member; V = volunteer; O = organiser. Figures in bold refer to a consistent distribution of respondents based on their social roles.

The comparison between these two solutions showed that 97.31% of respondents were distributed in a similar way whatever the method used (see Table 8).

**Table 8:** Cross-tabulation of the K-means and TwoStep solutions fixed at 5 clusters using z-scores from the first sub-sample.


K-MEANS	TWOSTEP					TOTAL

	NO	PASSIVE	LOW	MEDIUM	HIGH	

**no**	1317	14	0	0	0	1331

**passive**	0	820	68	15	0	903

**low**	0	0	868	0	10	878

**medium**	0	0	0	592	5	597

**high**	0	0	0	0	451	451

**Total**	1317	834	936	607	466	4160


*Note*. No = no social participation; passive = passive social participation; low = low active social participation; medium = medium active social participation; high = high active social participation. Figures in bold refer to a different distribution of respondents according to the clustering method used.

Among the differences observed, 68 respondents were distributed in the *passive* cluster using the K-means method and in the *low* cluster using the TwoStep method. The profile analysis (see Table 9) indicated that these 68 respondents had a social participation corresponding to the social roles *member & volunteer*. This profile description corresponded to the *low* active social participation cluster. As with the mean scores, the TwoStep solution proposed a more coherent clustering than that obtained with the K-means method.

**Table 9:** Description of profiles of 68 respondents grouped differently according to the clustering method used.


DIMENSIONS	N = 68	

	*M*	*SD*

**Breadth**	2.37	1.06

**Engagement M**	1.00	0.00

**Engagement V**	2.00	0.00

**Engagement O**	0.00	0.00

**Intensity Fre M**	4.87	1.13

**Intensity Fre V**	2.21	0.95

**Intensity Fre O**	0.00	0.00

**Intensity Vol M**	3.18	1.34

**Intensity Vol V**	1.99	0.87

**Intensity Vol O**	0.00	0.00

**Duration**	4.91	1.23


*Note*. Fre = frequency; Vol = hourly volume per month; M = member; V = volunteer; O = organiser. The scores shown are the average of the selected respondents.

**Figure 2:**
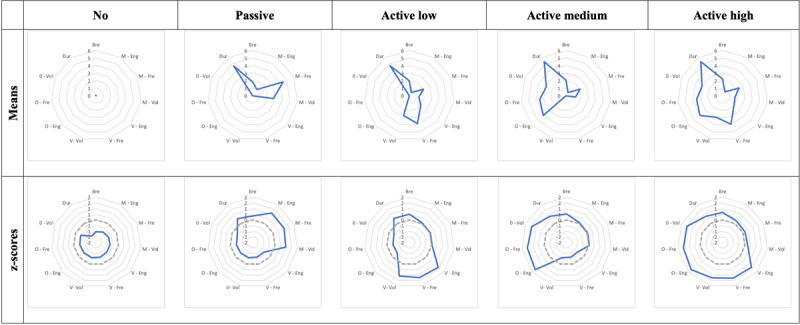
Selected clusters (TwoStep method) using the mean and standardised scores from the first sub-sample. *Note*. Bre = breadth; M = member; V = volunteer; O = organiser; Eng = engagement; Fre = intensity frequency; Vol = intensity volume; Dur = duration. Dashed lines are mean z-scores and solid lines are mean z-scores for each cluster.

At the end of these analyses, quite similar natural groups were observed whatever the method used (K-means vs. TwoStep) and the type of score used (mean vs. standardised). Figure 2 shows the clusters retained (TwoStep method) based on the mean and standardised scores from the first sub-sample.

### Discussion

Our proposed measure of social participation by clustering results in a ordinal variable that (a) reflects the degree of social participation by considering the four dimensions of the construct, (b) requires only six items and (c) is consistent with the way social engagement has been measured with the social role variable (MVO).

Our analyses using the social roles variable clearly showed the value of clustering and assessing all the dimensions proposed by Bohnert et al. (2010). In previous studies, researchers may have intuitively grouped all the organisers in the same cluster, whether they were also members or volunteers. However, our approach also considers the breadth, intensity, and duration of social participation in addition to engagement. In doing so, we discovered that organiser and volunteer respondents had similar rates of social participation to the high cluster respondents. Considering the four dimensions of social participation made it possible to create coherent and homogeneous groups.

Analysing social participation in this way provides a score by clustering which is limited to five levels and is easy to use in comparative analyses. Moreover, recoding the clusters (i.e., 0 = *no* social participation; 1 = *passive* social participation; 2 = *low active* social participation; 3 = *medium active* social participation; 4 = *high active* social participation) transforms the score into an ordinal variable that can be used in a Boolean format in regression and modelling analyses (e.g., SEM).

As the initial results met our expectations, we decided to replicate the analysis procedure with a second sample in a third study.

## Study 3

The purpose of the last study was to test the robustness of the procedure developed previously and, consequently, the clusterings obtained. In addition, Study 2 taught the reader how to perform clustering analyses. In Study 3, the reader will learn how to present the clustering results in a concise manner. The detailed procedure and all tables are available in the (see supplementary material available at https://osf.io/vumdw/?view_only=ffdac17d1e7e470aa78d699ee9b27cc6).

### Method

The second sub-sample consisted of 3,956 respondents with an average age of 57.20 years (SD = 17,434; min = 18; max = 94) and 59.3% women.

The measurements were the same as those used previously: social participation index and social roles (MVO).

### Results

**Figure 3:**
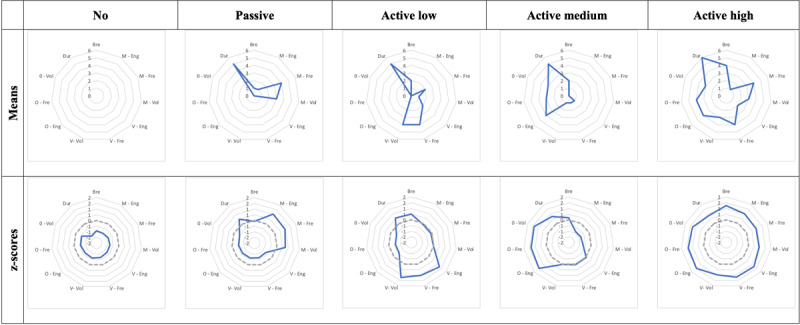
Selected clusters (K-means method) using the mean and standardised scores from the second sub-sample.

Following the instructions of the Social Participation Index, multivariate analyses were carried out to identify clusters of social participation in the data. First, hierarchical cluster analysis was carried out using Ward’s method with Euclidean squared distance measurement. The dendrogram suggested testing the 4-cluster and 5-cluster solutions. Secondly, successive non-hierarchical cluster analyses were carried out using K-means and TwoStep cluster analyses to identify the optimal solution. Moving from four to five clusters provided more homogeneous clusters and was preferred. The comparisons between the two 5-cluster solutions (i.e., K-means and TwoStep) showed that 86.98% of the respondents were classified in the same way regardless of the method used. Although the 5-cluster solutions were quite similar, K-means showed a more coherent allocation and was preferred. The same analyses were carried out using z-transformed variables. The second set of results was consistent with the previous set obtained using means. The selected profiles obtained with the TwoStep method using means and z-scores are illustrated in Figure 3.

### Discussion

The social participation index applied to another sample resulted in an ordinal categorical variable. The five new clusters obtained using the TwoStep method were similar to those previously obtained using the same method. However, applying the TwoStep method by default would have been contrary to the exploratory philosophy of multivariate clustering analysis.

By reproducing clustering analyses in their entirety, researchers can ensure that (a) clusters are not the result of chance but reflect trends that emerge in a consistent manner, and (b) the solution chosen is the most representative of the natural groups present in the data set. In the second subsample, the K-means method provided more consistent clustering than the TwoStep method and was preferred. Study 3 thus confirms the robustness of the process behind the social participation clusters we have identified.

## Conclusion

As was argued in the introduction, more and more researchers have adopted social participation as a variable in their surveys. However, there is a lack of consensus in the operationalisation of this variable. This prevents researchers from carrying out meta-analyses or comparing research results. To address this gap, the present article has discussed the development of a standard measure of social participation.

The first step was to look for a measure of social participation that took the four dimensions proposed by Bohnert et al. (2010) into account. By the end of the scoping review (Study 1), no measure that met this criterion had been found. In the absence of a satisfactory measure, we decided to develop one. The social participation index consists of a 6-item index that addresses both the recommendations of Bohnert et al. (2010) and the requirement that it can be used in large-scale surveys. This new measure was examined for quality in Study 2 and for replicability in Study 3 of this article. The score obtained by clustering analyses is a 5-level ordinal categorical variable which indicates the degree of social participation. Thus, the social participation score can be used for comparisons and can also be introduced in a Boolean format in regression and modelling analyses (Agresti, 2018). As a result, it should meet the needs of social science researchers who wish to assess social participation in large-scale surveys regardless of their discipline (e.g., psychology, sociology). It should be noted that researchers interested in specific questions aimed at improving clinical interventions around social participation can use the items separately. For example, the breadth item can be used to find out whether one type of activity should be favoured over another to benefit more from social participation. The use of future results in meta-analyses should be facilitated by the normative adoption of this new measurement tool by researchers.

Finally, some limitations are observed. First, the clustering is not yet normatively used in psychometrics. However, clustering is part of classical multivariate analysis in the same way as factor analysis or Cronbach’s alpha. In this sense, its application is well documented (e.g., Hair et al., 2010; James et al., 2013) and increasingly used in studies (e.g., Billieux et al., 2015; Bukowski et al., 2019). Its use was well suited to produce a consistent score from the social participation index. Clustering summarised information from items with different formats (e.g., dichotomous, Likert) while maintaining the precision of the four dimensions measured. In addition, the quality of the clusters identified is ensured by the full application of the analysis procedure. Second, the specific characteristics of the Belgian sample in partnership with Mutualité Chrétienne make it difficult to draw general conclusions. Application to other cultural or demographic contexts will be necessary to fully guarantee the generalisability of these initial results. Third, the Likert format of the items does not explore the qualitative aspects of social participation. Practitioners wishing to explore their patients’ social participation can use the index as a basis for a semi-structured interview to ensure that (a) the items are clearly understood and (b) qualitative experiences are captured.

In conclusion, this article paves the way for a multidimensional approach to the study of social participation. Such a four-dimensional approach provides a much richer theoretical context and a practical tool for understanding the impact of individual differences in social participation.
